# A phase IIb, open-label, randomized controlled dose ranging multi-centre trial to evaluate the safety, tolerability, pharmacokinetics and exposure-response relationship of different doses of delpazolid in combination with bedaquiline delamanid moxifloxacin in adult subjects with newly diagnosed, uncomplicated, smear-positive, drug-sensitive pulmonary tuberculosis

**DOI:** 10.1186/s13063-023-07354-5

**Published:** 2023-06-06

**Authors:** A Dierig, M Hoelscher, S Schultz, L Hoffmann, A Jarchow-MacDonald, EM Svensson, L Te Brake, R Aarnoutse, M Boeree, TD McHugh, LM Wildner, X Gong, PPJ Phillips, LT Minja, N Ntinginya, S Mpagama, A Liyoyo, RS Wallis, M Sebe, FA Mhimbira, B Mbeya, M Rassool, L Geiter, YL Cho, N Heinrich

**Affiliations:** 1 Division of Infectious Diseases and Tropical Medicine, LMU University Hospital Munich, Munich, Germany; 2 German Center for Infection Research (DZIF), Munich partner site, Munich, Germany; 3 Ninewells Hospital and Medical School, NHS Tayside, Dundee, UK; 4 Department of Pharmacy, Radboud University Medical Centre, Nijmegen, The Netherlands; 5 Department of Pharmacy, Uppsala University, Uppsala, Sweden; 6 Division of Infection & Immunity, UCL Centre for Clinical Microbiology, University College of London, London, UK; 7 Department of Medicine, Division of Pulmonary & Critical Care Medicine, University of California San Francisco, San Francisco, USA; 8 National Institute for Medical Research, Mbeya Medical Research Centre (NIMR-MMRC), Mbeya, Tanzania; 9 Kilimanjaro Clinical Research Institute, Kilimanjaro, Tanzania; 10 The Aurum Institute, Tembisa, South Africa; 11 Ifakara Health Institute, Dar es Salaam, Tanzania; 12 Clinical HIV Research Unit, Department of Internal Medicine, School of Clinical Medicine, Faculty of Health Sciences, Helen Joseph Hospital, University of the Witwatersrand, Johannesburg, South Africa; 13 LegoChem Biosciences, Daejeon, South Korea

**Keywords:** Uncomplicated pulmonary tuberculosis, Treatment, Delpazolid, Randomized controlled trial, Phase IIb, Oxazolidinone

## Abstract

**Background:**

Linezolid is an effective, but toxic anti-tuberculosis drug that is currently recommended for the treatment of drug-resistant tuberculosis. Improved oxazolidinones should have a better safety profile, while preserving efficacy. Delpazolid is a novel oxazolidinone developed by LegoChem Biosciences Inc. that has been evaluated up to phase 2a clinical trials. Since oxazolidinone toxicity can occur late in treatment, LegoChem Biosciences Inc. and the PanACEA Consortium designed DECODE to be an innovative dose-ranging study with long-term follow-up for determining the exposure–response and exposure–toxicity relationship of delpazolid to support dose selection for later studies. Delpazolid is administered in combination with bedaquiline, delamanid and moxifloxacin.

**Methods:**

Seventy-five participants with drug-sensitive, pulmonary tuberculosis will receive bedaquiline, delamanid and moxifloxacin, and will be randomized to delpazolid dosages of 0 mg, 400 mg, 800 mg, 1200 mg once daily, or 800 mg twice daily, for 16 weeks. The primary efficacy endpoint will be the rate of decline of bacterial load on treatment, measured by MGIT liquid culture time to detection from weekly sputum cultures. The primary safety endpoint will be the proportion of oxazolidinone class toxicities; neuropathy, myelosuppression, or tyramine pressor response.

Participants who convert to negative liquid media culture by week 8 will stop treatment after the end of their 16-week course and will be observed for relapse until week 52. Participants who do not convert to negative culture will receive continuation phase treatment with rifampicin and isoniazid to complete a six-month treatment course.

**Discussion:**

DECODE is an innovative dose-finding trial, designed to support exposure-response modelling for safe and effective dose selection. The trial design allows assessment of occurrence of late toxicities as observed with linezolid, which is necessary in clinical evaluation of novel oxazolidinones. The primary efficacy endpoint is the change in bacterial load, an endpoint conventionally used in shorter dose-finding trials. Long-term follow-up after shortened treatment is possible through a safety rule excluding slow-and non-responders from potentially poorly performing dosages.

**Trial registration:**

DECODE was registered in ClinicalTrials.gov before recruitment start on 22 October 2021 (NCT04550832).

## Administrative information

Note: The numbers in curly brackets in this protocol refer to SPIRIT checklist item numbers. The order of the items has been modified to group similar items (see http://www.equator-network.org/reporting-guidelines/spirit-2013-statement-defining-standard-protocol-items-for-clinical-trials/). The items from the trial registry are found within the protocol.

**Table:** 

Title	A phase IIb, open-label, randomized controlled dose ranging multi-center trial to evaluate the safety, tolerability, pharmacokinetics and exposure-response relationship of different doses of delpazolid in combination with bedaquiline delamanid moxifloxacin in adult subjects with newly diagnosed, uncomplicated, smear-positive, drug-sensitive pulmonary tuberculosis Trial acronym: DECODE (PanACEA DElpazolid dose-finding and COmbination DEvelopment)
Trial registration {2a and 2b}.	NCT04550832 16/09/2020 Clinicaltrials.gov
Protocol version {3}	Version 2.1 – 08.09.2021
Funding {4}	This trial is funded by LegoChem Biosciences. LegoChem Biosciences will also supply the treatment with delpazolid.
Author details {5a}	^1^LMU University Hospital Munich, Division of Infectious Diseases and Tropical Medicine, Munich, Germany ^2^German Center for Infection Research (DZIF), Munich partner site, Munich, Germany ^3^Ninewells Hospital and Medical School, NHS Tayside, Dundee, UK ^4^Department of Pharmacy, Radboud University Medical Centre, Nijmegen, The Netherlands ^5^Department of Pharmacy, Uppsala University, Uppsala, Sweden ^6^UCL Centre for Clinical Microbiology, Division of Infection & Immunity, University College of London. UK. ^7^University of California San Francisco, Department of Medicine, Division of Pulmonary & Critical Care Medicine, San Francisco, United States ^8^National Institute for Medical Research – Mbeya Medical Research Centre (NIMR-MMRC), Mbeya, Tanzania ^9^Kilimanjaro Clinical Research Institute, Kilimanjaro, Tanzania ^10^The Aurum Institute, Tembisa, South Africa ^11^Ifakara Health Institute, Dar es Salaam, Tanzania ^12^Clinical HIV Research Unit, Helen Joseph Hospital, Department of Internal Medicine, School of Clinical Medicine, Faculty of Health Sciences, University of the Witwatersrand, South Africa ^13^Consultant, LegoChem Biosciences, South Korea ^14^LegoChem Biosciences, South Korea
Name and contact information for the trial sponsor {5b}	LegoChem Biosciences, Inc. 10, Gukjrgwahak 10-ro, Yuseong-gu Daejeon Republic of Korea
Role of sponsor {5c}	The trial sponsor will have the ultimate decision on trial design, data collection, data management, analysis, and interpretation of data, writing of the clinical study report, and the decision to publish the clinical study report. The PanACEA Consortium authors were contracted by the sponsor to advise on the activities above. PanACEA authors have a contractually agreed freedom to publish study results; the sponsor has the right to comment on manuscripts prior to publication.

## Introduction

### Background and rationale {6a}

Tuberculosis (TB) is the thirteenth leading cause of death worldwide and, before the advent of COVID-19, was the leading cause from a single infectious agent, ranking above human immunodeficiency virus (HIV)/AIDS [1]. It remains a persistent problem, especially in the developing countries of Africa, Asia and Eastern Europe. The current first-line anti-tubercular agents have been in use for over 50 years and are ineffective in controlling TB as a public health problem. The long treatment duration and treatment-related toxicity result in poor compliance and as a result, drug resistance is becoming more common. Multidrug-resistant TB (MDR-TB) is a public health emergency, especially in sub-Saharan Africa, where HIV infection is endemic. Prevailing challenges such as lack of universal MDR-TB diagnosis, lengthy and toxic treatments that cure only 52% of participants, complicated with development of extensively drug-resistant TB (XDR-TB), are major impediments in MDR-TB control. A mathematical epidemiological model of MDR-TB in Vietnam showed that under current diagnostic and treatment practices, MDR-TB incidence will increase by 17%, and deaths by 22%, within ten years [2]. In a similar model in China, MDR-TB was predicted to become the dominant form of TB by 2050 [3].

Linezolid (LZD), an oxazolidinone, approved by the US Food and Drug Administration (FDA) since 2000 for the treatment of Gram+ infections, acts to inhibit protein synthesis by binding the 23S ribosomal RNA (rRNA) portion of the bacterial 50S subunit.

LZD has shown good efficacy in randomized controlled trials (RCTs) and cohort studies, mostly conducted in XDR-TB patients. In a landmark study conducted by Lee et al., LZD was added to failing treatment in participants with extensive resistance; a strategy which is otherwise not advised since the absence of active combination partner drugs predisposes towards new resistance against the drug that is added [4–6]. The Lee study, however, reported a culture conversion rate of 87% after 6 months of LZD treatment, at a median of 75 days after the start of treatment with LZD. Overall treatment success one year after treatment end was achieved in 27 of 38 participants receiving LZD. Four participants suffered treatment failure with acquired LZD resistance, 4 more withdrew, and 3 were lost to follow-up. These excellent efficacy numbers are in line with a meta-analysis of the case series, reporting 79.5 % of M/XDR TB patients achieving favourable outcomes with LZD [7].

In the NiX-TB trial, a novel regimen composed of LZD, bedaquiline (BDQ) and pretomanid (PTM) achieved cure in 90% of participants with XDR-TB or failing MDR-TB treatment [8].

LZD has a challenging safety profile, and its use is limited by adverse events (AEs) due to its inhibition of mitochondrial protein synthesis. Given long-term toxicities related to LZD treatment are anaemia, neutropenia, thrombocytopenia, peripheral and optic neuropathy. In addition, the inhibition of monoamine oxidase A (MAO A) by LZD may result in hypertensive responses after dietary intake of tyramine (tyramine pressor response, “cheese syndrome”) [9].

In NiX-TB, only 15% of participants completed the 6-month course of LZD without interruptions or dose reductions due to toxicity. The Global TB Alliance then set up a ZeNix trial to evaluate whether shorter dosing of LZD or dosing at a lower dose would achieve an acceptable toxicity while maintaining efficacy. This approach was partially successful, but still at the lowest dose tested for the shortest duration (600mg for 9 weeks), 6/45 (13.3%) of participants developed neuropathy (M.Olugbosi, presented at InterTB Meeting 2021); illustrating the need for safer novel oxazolidinones.

The Pan-African Consortium for the Evaluation of Antituberculosis Antibiotics (PanACEA; http://panacea-tb.net/) is conducting two trials to evaluate novel oxazolidinones; delpazolid (DZD) developed by LegoChem Biosciences (this trial, DECODE), and sutezolid (STZ), developed by Pfizer/Sequella (the SUDOCU trial, ClinicalTrials.org identifier. NCT03959566).

The new oxazolidinones are combined with Delamanid (DLM), a nitroimidazole, and will be used in combination with BDQ and Moxifloxacin (MXF). Safety data suggests that these drugs may have a more favourable safety profile compared to their counterparts LZD and PTM. DLM is approved for use in Europe, Japan, and several other countries. DZD is a new, investigational oxazolidinone that is anticipated to have similar or better efficacy, compared to LZD and may cause less toxicity than LZD. Nonclinical studies demonstrated that DZD is not metabolized by major cytochrome P450 enzymes nor does it inhibit any of them. Also, DZD is neither perpetrator nor victim of drug-drug interactions based on major transporters. Prior to the study, DZD had been studied in 161 healthy participants in Phase 1 studies with the longest treatment being 21 days. 45 subjects were treated for 14 days in an early bactericidal activity (EBA) monotherapy study, but no reliable dose-response was identified for efficacy or toxicity. To assess the safety and efficacy of different doses of DZD, a trial design needs to be chosen that permits longer exposure to an oxazolidinone as most toxicities occur after an exposure of 14 days. Therefore, we developed a trial design that permits longer exposure through combination therapy with established anti-tuberculosis agents. Data from this study will inform the selection of a DZD dose for further assessment in the following studies.

### Objectives {7}

The primary objective of this study is to generate data for a pharmacokinetic–pharmacodynamic modelling approach, and to establish the exposure–response and exposure–toxicity curve for DZD.

The aim is to identify the optimal dose of DZD to be used in subsequent studies that will provide the best efficacy and acceptable safety profile for the drug when given daily over 16 weeks in participants with newly diagnosed, uncomplicated, smear-positive, drug-sensitive, pulmonary tuberculosis.

This will be supported by the development of a population pharmacokinetic (PK) model.

Importantly, DECODE will assess the proportion of participants who suffer relapse within 12 months post randomization, out of those participants completing 16 weeks of therapy and achieving sustained sputum culture conversion, defined as two successive negative liquid media (BD BACTEC™ MGIT ) cultures at or before week 8, with no positives to follow until the week 16 visit.

Secondary efficacy objectives are other culture-based response metrics, including month-2 culture status in liquid media and on solid media, and time to culture conversion in liquid and on solid media.

The secondary PK objective is to describe the PK of BDQ, DLM and MXF including their main metabolites.

In addition, this study has mycobacteriological identification and characterization objectives, which are (i) to assess the minimum inhibitory concentrations of BDQ, DLM, MXF, and DZD of the infecting strain (ii) to investigate the frequency of acquired mutations in the infecting strain over treatment and (iii) to compare the initial and recurrence isolates in participants with recurrent disease by whole genome sequencing to discriminate relapse from reinfection.

### Trial design {8}

This will be an open-label, phase IIb, randomized, controlled, dose-finding, multi-centre study in participants with newly diagnosed, smear-positive, uncomplicated drug-sensitive pulmonary TB. Adult participants (≥ 18 years of age) will be randomized by centralized allocation at a ratio of 1:1:1:1:1 to one of five treatment arms containing BDQ, DLM and MXF, combined with different doses of DZD.

After the completion of 16 weeks of experimental treatment, participants in the experimental arms, who did not achieve two successive negative liquid media cultures with the first at or before week 08, with no following positive results reported by the week 16 visit, will be referred to their local health care facility to complete their course of anti-TB treatment according to the national TB program. All other participants will be followed up until week 52 to rule out relapse or re-infection.

## Methods: participants, interventions and outcomes

### Study setting {9}

The study will be implemented in South Africa and Tanzania, with enrolment taking place in two dedicated TB trial centres in Johannesburg, South Africa, and in three dedicated TB trial institutes in Mbeya, Dar es Salaam and Moshi, Tanzania. A list of all participating study centres can be obtained at www.ClinicalTrials.gov. The burden of drug-sensitive TB is high in Tanzania with an estimated incidence of all forms of TB of 222 per 100000 in 2020 [1]. The proportion of notified MDR/RR (Rifampicin resistant)-TB cases among all notified new cases of TB remains low, at 0.5 [1]. South Africa has one of the highest TB burden globally with an incidence of 554 per 100000 in 2020. The rate of notified MDR/RR-TB of all notified cases is 3.3% [1]. The rate of HIV-positive participants (among all participants with known HIV status) among participants tested for TB is 21% in Tanzania and 47% in South Africa. HIV is known to be a major risk factor for TB and at least in South Africa one of the biggest contributors of the TB epidemic.

### Eligibility criteria {10}

Participants will be enrolled if they fulfil all of the following criteria:

1. Provide written, informed consent prior to all trial-related procedures
2. Aged between 18 and 65 years
3. Body weight between 40 and 90 kg
4. Are newly diagnosed with RIF and INH drug-susceptible TB
5. Have a chest x-ray consistent with TB
6. Are sputum positive on microscopy from concentrated sputum for acid fast bacilli on at least one sputum sample (at least 1+ IUATLD/WHO scale)
7. Show willingness to forgo consumption of foods high in tyramine
8. Participant or partner are unable to conceive/father children and/or using effective methods of contraception.

Participants will be excluded if they meet any of the following criteria:

1. Are pregnant or breastfeeding
2. Infection with HIV with a CD4 count <220 cells/mm^3^. If >220 cells/mm^3^, participants will be included only if *any* of the following is applicable:
  The participant is antiretroviral (ARV) naïve and able to postpone commencing HIV treatment for 2 months after the trial has started and then restrict regimens to those containing dolutegravir
    *or*
    The participant is ARV experienced (has been on ARV´s a minimum of 5 months) and able to switch to a dolutegravir-based regimen.
  The participant is treated with nucleosidic reverse transcriptase inhibitors (are permitted as concomitant medication).
  The participant is treated with protease inhibitors as part of antiretroviral treatment (ART) regimens, which will be stopped at least 3 days before the start of study treatment (WK00, day1) for a participant to be eligible.
  The participant is treated with Efavirenz as part of ART regimens which would have to be stopped 14 days before the start of study treatment (WK00, day 1) for a participant to be eligible.
3. Known intolerance to any of the study drugs or concomitant disorders or conditions for which study drugs or standard TB treatment are contraindicated.
4. History of, or current evidence of clinically relevant cardiovascular metabolic, gastrointestinal, neurological, psychiatric or endocrine diseases, malignancy, or any other condition that will influence treatment response, study adherence or survival in the judgement of the investigator, especially:
  a Neuropathy, or significant psychiatric disorder like depression or schizophrenia; especially if treatment for those has ever been required or is anticipated to be required
  b Clinically significant evidence of extra-pulmonary TB (e.g. miliary TB, TB meningitis, but not limited lymph node involvement)
  c Serious lung conditions other than TB, or significant respiratory impairment in the discretion of the investigator
  d Any diabetes mellitus
  e Cardiovascular disease such as myocardial infarction, heart failure, coronary heart disease, arrhythmia, tachyarrhythmia, or pulmonary hypertension
  f Arterial hypertension (systolic BP ≥140 mmHg and/or diastolic BP of ≥90 mmHg on two occasions during screening)
  g Long QT syndrome or family history of long QT syndrome or sudden death of unknown or cardiac-related cause
  h Alcohol or other drug abuse that is sufficient to significantly compromise the safety or cooperation of the participant, that includes substances prohibited by the protocol or has led to significant organ damage at the discretion of the investigator.
5. Any of the following laboratory findings at screening:
  a Serum amino aspartate transferase (AST) and/or alanine aminotransferase (ALT) >3× the upper limit of normal (ULN)
  b Serum alkaline phosphatase or y-glutamyl transferase > 2.5× the ULN
  c Serum total bilirubin level >1.5× the ULN
  d Estimated creatinine clearance (eCrCl) < 30 ml/min
  e Serum albumin < 2.8 g/dl
  f Haemoglobin level <7.0 g/dl
  g Platelet count <50,000/mm^3^,
  h Serum potassium below the lower level of normal (laboratory specific)
  i Blood glucose at screening < 70mg/dL (3.9mmol/L)
6. Electrocardiogram (ECG) findings in the screening ECG: (one or more):
  a Fridericia corrected QT interval (QTcF) of >0.450 s
  b Atrioventricular (AV) block with PR interval > 0.20 s
  c QRS complex > 120 ms
  d Any other changes in the ECG that are clinically relevant as per discretion of the investigator
7. Restricted medication:
  a Treatment with any other investigational drug within 1 month prior to enrolment or enrolment into other clinical (intervention) trials during participation.
  b Previous anti-TB treatment with drugs active against *Mycobacterium tuberculosis* (MTB) within the last 3 months prior to screening.
  c Unable or unwilling to abide by the requirements regarding restricted medication or have taken restricted medication. Restricted medication includes the following drug classes:
    Anti-TB drugs other than study drugs
    Medication that lowers the threshold for epileptic seizures
    Medication that prolongs the QTc interval
    Drugs that affect monoamine oxidase (MAO) or serotonin metabolism
    CYP 450 inhibitors or inducers, including grapefruit- containing foods / beverages and St. John’s Wort

### Who will take informed consent? {26a}

Information about the trial is provided by study doctors/nurses at the trial site, who need to be delegated to perform these tasks. Participants will be invited to be screened for inclusion in the trial if they are suspected to have pulmonary TB or have an established diagnosis by smear microscopy, GeneXpert or chest X-ray done within the government or private health sector. The investigator or a person designated by the investigator will inform the participants or the participant’s legally acceptable representative in detail. After signing the informed consent form, participants are screened for inclusion and exclusion criteria, and, if eligible, enrolled in the study and randomized to one of the treatment arms.

### Additional consent provisions for collection and use of participant data and biological specimens {26b}

The study aims to collect further data and biological samples to advance the science around TB and TB treatment. Therefore, additional informed consent will be thought to collect retention samples and samples for genetic analysis for possible sub-studies.

## Interventions

### Explanation for the choice of comparators {6b}

This dose-finding study is small and does not contain a classical comparator arm. 15 participants will receive DLM, BDQ, and MOX without DZD, but this is not powered to show the effect of adding DZD, unless the core regimen or DZD is much more effective or much less effective than anticipated, although this arm does provide data on a ‘0mg’ dose of DZD for the exposure modelling analysis. The intention is to provide data to set up an exposure-response model for DZD on the basis of a backbone regimen composed of the 2^nd^-line TB drugs BDQ, DLM and MXF, at licensed doses: BDQ 400mg once daily for 14 days, then 200mg thrice weekly; DLM 100mg twice daily, and MXF 400mg once daily.

BDQ, a diarylquinoline compound, is the first new anti-TB drug approved after 40 years by the FDA, also specifically, as part of a combination therapy for the treatment of MDR-TB. It is approved and part of the national standard recommended treatment regimen for RR- and MDR-TB in South Africa [10]. It is also approved in Tanzania. Furthermore, BDQ has been recommended as part of MDR-TB treatment by the WHO [11].

DLM, a nitroimidazole, represents a promising new drug for the treatment of MDR-TB. It has received regulatory approvals in several countries and has been recommended by the WHO for the treatment of MDR-TB in specific cases [11, 12].

MXF is a fluoroquinolone (FQ). FQ are a mainstay of MDR-TB treatment and MXF, is considered as one of the most potent drug in second-line MDR-TB therapy as recently reviewed by the WHO. Furthermore, MXF has an excellent safety profile according to data on its long-term use.

### Intervention description {11a}

**Fig. 1:**
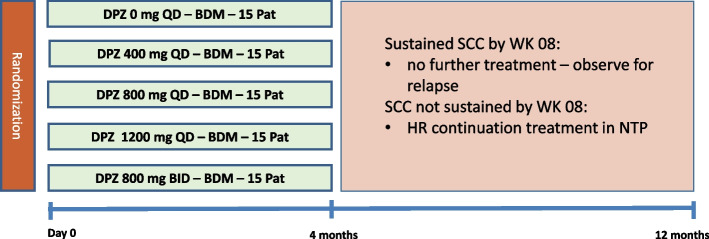
Study design and five different treatment arms. DZD, delpazolid; BDM, bedaquiline, delamanid, moxifloxacin; QD, once daily; BID, twice daily; SCC, sustained culture conversion to negative; WK08, treatment week 08; HR, isoniazid – rifampicin; NTP, national TB programme

After assessing the eligibility of the participants at screening, the study will recruit into five different treatment arms. The experimental and control treatment will be administered daily for 16 weeks. Figure 1 gives an overview of the different treatment arms. All anti-TB drugs have to be taken with food (standardized only at WK02 visit for intensive PK sampling) and a glass of water.

### Criteria for discontinuing or modifying allocated interventions {11b}

Besides withdrawal of consent, requiring medication prohibited by protocol or sponsor decision, several stopping criteria for participants’ safety are in place:

• *Hepatotoxicity stopping criteria*: these follow the FDA guidance on evaluation of investigational drugs for causation of drug-induced liver injury [13].

A participant should discontinue study drug if:

- ◦ ALT or AST >8×ULN
- ◦ ALT or AST >5×ULN for more than 2 weeks
- ◦ ALT or AST >3×ULN and (total bilirubin (TBL) >2×ULN or INR >1.5
- ◦ ALT or AST >3×ULN with the appearance of fatigue, nausea, vomiting, right upper quadrant pain or tenderness, fever, rash, and/or eosinophilia (>5%)
• *ECG stopping criterion*: Participants will stop treatment if their on-treatment ECG shows a prolongation of the QTcF interval on average in triplicate ECGs to grade 3 as defined in Table 1.

**Table 1:** severity grading for the QTcF interval on-treatment ECGs, adapted from [14]

Grade 1	Grade 2	Grade 3	Grade 4	Grade 5
(1) Absolute QTcF >480 and ≤500 ms and QTcF change from baseline >0 ms and ≤30 ms; or	(1) Absolute QTcF >480 ms and ≤500 ms and QTcF change from baseline >30 ms and ≤ 60 ms; or	(1) Absolute QTcF >500 ms; or	Life-threatening consequence, e.g. torsades de pointes or other associated serious ventricular dysrhythmia.	-
(2) absolute QTcF ≤480 ms and QTcF change from baseline >30 and ≤60 ms	(2) absolute QTcF ≤480 and QTcF change from baseline >60 ms.	(2) absolute QTcF >480 and QTcF change from baseline >60 ms.		-
• *Neuropathy stopping criterion*: Participants will stop treatment with DZD in case they develop clinically significant signs of motor or sensory neuropathy, i.e. loss of muscle strength, loss of sensation, loss of vibration sensitivity, or loss of visual acuity or colour vision.
• *Hypertension (tyramine pressor effect) stopping criterion:*

BP systolic >160 mmHg, or diastolic >100 mmHg:

- ◦ Re-assessment: Participants who develop significant hypertension with systolic blood pressure (BP) averages of three measurements of ≥ 160 mm Hg, and/or diastolic BP of ≥ 100 mm Hg, but less than 180/110 mmHg, will be re-assessed on 2 separate occasions.
- ◦ They should be re-counselled as to the foods and drink to be avoided with study treatment, as non-compliance to this could be an important aspect to the hypertension.
- ◦ If this evaluation supports the conclusion of a significant increase in blood pressure, the investigator will assess potential causes. If the increase is determined to be associated with study treatment, a de-challenge/re-challenge will be performed: participants will discontinue DZD. If, after ≥ 10 h (> 5 × t_1/2_ of DZD) after the last dose, BP has dropped significantly, a re-challenge with daily BP measurements will be considered.
- ◦ Treatment with BDQ, DLM and MXF should continue throughout.
- ◦ Continued hypertension after re-assessment: participants who develop persistent hypertension ≥ 160/100 mmHg after evaluation and adequate antihypertensive treatment, including those who have undergone a re-challenge with DZD, will discontinue all study treatment and complete TB treatment according to national TB program guidelines. These participants will receive follow-up to determine whether the condition normalizes after discontinuation of study treatment.

BP systolic >180 mmHg, or diastolic >110 mmHg:

- ◦ Immediate stop**:** Participants who develop hypertension with systolic BP averages of three measurements of ≥ 180 mm Hg, and/or diastolic BP of ≥ 110, will stop study treatments immediately, and receive antihypertensive treatment. During follow-up, investigators should attempt to determine whether the condition normalizes after discontinuation of study treatment, in order to better judge relatedness to IMP.
• *Serotonin syndrome stopping criterion*: A participant will stop treatment with DZD if at least one of the following criteria are fulfiled, which are indicative of serotonin syndrome (Hunter Serotonin Toxicity Criteria [15]:
- ◦ Spontaneous clonus
- ◦ Inducible clonus PLUS agitation or diaphoresis
- ◦ Ocular clonus PLUS agitation or diaphoresis
- ◦ Tremor PLUS hyperreflexia
- ◦ Hypertonia PLUS temperature above 38°C PLUS ocular clonus or inducible clonus
• *Convulsions/seizures stopping criterion*: A participant will stop study treatment if clinically significant convulsions are observed in the discretion of the investigator.

### Strategies to improve adherence to interventions {11c}

Study treatment intake will be observed by study staff during the study visits in the morning and will be administered at home on the other days. Facility-based directly observed treatment or community-based directly observed treatment (i.e. a friend or relative of the participant will act as a treatment supervisor) will be in place in order to maximize adherence. Furthermore, treatment adherence will be assessed by pill counting at every visit.

### Relevant concomitant care permitted or prohibited during the trial {11d}

BDQ, DLM and MXF are metabolized by different hepatic enzymes (CYP3A4-BDQ and DLM; UDP-glucuronoyltransferase-MXF). A change in activity of these hepatic enzymes can change the drug concentrations in blood and tissue and thereby influence safety and efficacy readouts. Therefore, all drugs that would lead to a substantial change in activity of these enzymes are prohibited. Participants who are already enrolled and on study medication, and a need arises to treat with any of those drugs during the treatment phase of the trial, experimental treatment may have to be stopped.

In addition, several other classes of drugs are prohibited during the trial as they might interfere with the assessment of possible AEs of the drugs given during the trial. These drugs include drugs acting on the MTB complex, drugs that might induce epileptic seizures by lowering the threshold, drugs potentially prolonging the QT-interval, drugs affecting the MAO, serotonin agonists and CYP450 inducer or inhibitor. Also, as oxazolidinones are known to have a weak reversible MAO inhibitory effect in vitro, they can block the metabolization of dietary tyramine and thus might act as pressor enhancers [9]. This is documented for LDZ in rats and in rare cases in humans. As no studies so far were conducted with DZD to address this issue, participants will be asked to avoid food rich in tyramine. Special considerations are taken into account for HIV-positive participants on ART: efavirenz and protease inhibitors are not permitted due to potential effects on the TB drugs via CYP3A4 inhibition or induction. Dolutegravir is the third drug of choice to complete an ARV regimen, together with two nucleosidic reverse transcriptase inhibitors.

Additionally, all participants able to conceive/father children will consent to be using two effective methods of contraception, one of which must be a barrier method. These will be explained to the participants in detail. Serum pregnancy tests in all females of childbearing potential will be taken at screening, week 09 and week 18 visit.

### Provisions for post-trial care {30}

Participants who achieve two successive negative liquid media cultures, the first of which is at or before week 08, with no positives to follow by the week 16 visit, will not receive further standard of care TB-treatment (continuation phase) according to national guidelines to complete 6 months of treatment. Their planned post-treatment follow-up visits at week 18, week 26, week 38 and week 52 will serve to determine whether they have achieved lasting cure. Participants who do not fulfil these criteria will receive standard of care TB-treatment according to national guidelines until week 26 at a government health facility. These participants will be invited to return for a follow-up visit at week 52 to determine their well-being and treatment outcome.

Clinical trial insurance is obtained to compensate participants in case participating in this study causes any harm.

### Outcomes {12}

The primary safety outcome is the occurrence of oxazolidinone class toxicities defined as peripheral or optical neuropathy, incident leukopenia, anaemia or thrombocytopenia, or AEs in line with tyramine pressor response, all of grade 2 or higher, possibly, probably or definitely related to DZD. Participants will be evaluated for AEs on a regular basis during treatment and follow-up phase.

The efficacy of DZD will be evaluated by measuring the change in mycobacterial load over time on treatment as quantified by time to positivity in BD BACTEC™ MGIT liquid culture described by non-linear mixed-effects methodology.

A secondary endpoint is the proportion of participants who suffer relapse, defined as recurrent disease caused by a strain identical to the baseline isolate, within 12 months post randomization, out of participants completing 16 weeks of therapy and achieving sustained sputum culture conversion defined as two successive negative liquid media cultures at or before WK08, with no positives to follow by the week 16 visit. We will analyze this also by a time-to event analysis (time to recurrent TB, and to relapse). Pharmacokinetic endpoints are chosen to support the development of a population PK model for DZD. In addition, we will use non-compartmental analysis for DZD, BDQ, DLM and their main metabolites, and for MXF, to determine the area under the plasma concentration curve from 0 to 24 h on day 14, the observed maximum concentration, the time of maximum concentration and the minimum observed plasma concentration. Furthermore, the apparent oral clearance, apparent volume of distribution and terminal half-life will be determined for MOX only.

Mycobacteriological Identification and Characterization Endpoint:

• Isolates will be assessed for minimum inhibitory concentrations of BDQ, DLM, MXF, and DZD of the infecting strain, at baseline and on representative isolate(s) grown at or after WK08, if any.
• In the case of recurrent disease, a comparison between bacterial strain causing recurrent disease, and the strain at baseline will be performed by whole genome sequencing, to discriminate relapse from re-infection.

### Participant timeline {13}

**Fig. 2:**
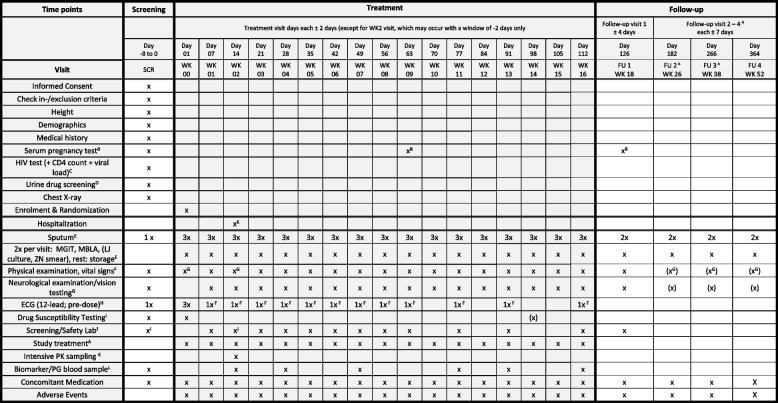
Schedule of events. WK, week of treatment; MGIT, liquid media (BD mycobacterium growth indicator tube); LJ, Loewenstein - Jensen solid media; MBLA, molecular bacterial load assay; PK, pharmacokinetics; X, refers to all visits mentioned above; ZN, Ziehl-Neelsen stain; PG, pharmacogenomics

The timeline of the study (see Fig. 2: schedule of events, modified SPIRIT figure template) is divided into three parts: (i) screening to confirm which participant is eligible for the study, (ii) treatment according to the allocated randomization for a total of 16 weeks, with weekly visits and 2 sputum samples obtained for culture per visit, and (iii) follow-up until week 52. At every weekly treatment phase visit, ECGs are registered, safety laboratory samples obtained, physical and neurological examination performed including visual acuity examination. At the week 2 visit, participants are hospitalized to perform intensive PK sampling.

### Sample size {14}

Fifteen participants per arm with a total of 75 participants and a wide range of DZD doses (from 0mg to 800 mg BID) has been determined as an adequate sample size for population PK modelling, and for exposure-response modelling to detect a clinically meaningful dose-dependent relationship.

Furthermore, the planned sample size of 15 participants per treatment group is in keeping with other trials of this type and accounts for the possibility of up to 3 drop-outs per group, which, based on previous studies of this type conducted at these sites, represents a conservative estimate of the expected drop-out rate.

Previous Phase IIA (EBA) studies indicate that the between-participant standard deviation of logCFU can be approximately 0.2 [16]. Therefore, assuming similar variability in this trial the expected standard errors of group mean EBA and corresponding width of 95% confidence intervals are 0.052 and 0.101 respectively for a group size of 15 and 0.063 and 0.124, respectively, for a group size of 10. This level of precision with a group size of 15 is considered adequate.

### Recruitment {15}

Sites will place recruitment teams in government health clinics where TB diagnostics are offered. Participants who test positive will be informed about the trial and invited for screening.

Recruitment can be improved by individual and community awareness of the study and/or TB in general, through public announcements through advertisements, posters and radio announcements and information leaflets distributed to healthcare providers for their participants with newly diagnosed TB.

## Assignment of interventions: allocation

### Sequence generation {16a}

The study will be a randomized, open-label trial. Randomization will be implemented after all screening results are available for participants who have given informed consent and who have been found eligible for participation.

Participants will be allocated using the Internet-Based Randomization service system: RANDOMIZE.NET. Participant randomization will be stratified by a bacterial load in sputum as measured by GeneXpert, cycle threshold (≥ 16, < 16), site (five sites have been activated: The Aurum site, Clinical HIV Research Unit (CHRU), KCRI/KIDH, the Ifakara site and the NIMR-MMRC site), and HIV status (positive, negative). Each site will have its own account and the allocation result will be generated by the web system immediately based on a minimization randomization algorithm. The minimization algorithm allocates the participant to the treatment arm with the lowest allocation proportion and includes a probabilistic element so that the allocation is not deterministic.

### Concealment mechanism {16b}

The allocation sequence is generated by a web-based randomization system set up by the sponsor, with the investigator entering patient details. A minimization algorithm with a random element generates the treatment allocation; the random element prevents the investigator from knowing the allocation before the randomization process.

### Implementation {16c}

The web-based randomization system has been set up by the sponsor statistician on the Internet-Based Randomization service system: RANDOMIZE.NET. Participants will be enrolled by the allocated study staff (investigator). After all screening results are available and eligibility is proven, the investigator will request a treatment allocation from the system for the individual participant in question. Allocation concealment will be granted by the inbuilt random element.

## Assignment of interventions: blinding

### Who will be blinded {17a}

The personnel assessing participants’ outcomes, like the microbiology laboratory staff or the sponsor medical expert discussing the possibility of recurrent disease, will remain blinded to treatment assignment throughout the whole study in order to ensure unbiased assessment of efficacy endpoints, in attribution of AE causality and expectedness, and in discussion on management with site staff. This is laid down in the trial protocol and associated documents; and data fields from the study database that show treatment assignment, will not be shared with those persons before formal database lock. The data analyst will not be blinded, which is required for composing unblinded reports to the DSMB.

### Procedure for unblinding if needed {17b}

In the unlikely event that unblinding is necessary in the interest of participants’ safety and well-being throughout the study for sponsor, sponsor medical expert and other blinded staff, this will be requested from the unblinded statisticians and documented.

## Data collection and management

### Plans for assessment and collection of outcomes {18a}

For this study, a data management group created a predefined data base. On a weekly basis, the sponsor receives data reports for review, including listings of blood results for the safety management of the participants. These listings help to identify and to act upon weaknesses in data capture but also quality. Further, this group will send queries to the responsible site in case data has been entered incorrectly or is missing. Several manuals (e.g. lab manual, manual of procedure for clinical assessment of the participants, including “red flags” for discussion with the sponsor medical expert, PK manual) exist to promote data quality. A site initiation visit will be conducted prior to study start to train assessors and a re-training will be conducted in case changes of the protocol occur.

Further, time to detection (TTD) is a measurement of bacterial load in the liquid culture BD MGIT system. In order to reduce variability, we will collect two sputum samples per weekly visit and inoculate a MGIT culture from each sample.

### Plans to promote participant retention and complete follow-up {18b}

The process of promoting retention will begin at consent by building a trusting relationship between the participant and the clinical team, as participants are more likely to adhere to study schedules if they know from the outset what they are agreeing to. The study team will collect participants’ demographic information including mobile phone contact(s) and physical address. Using the study visit calculator, participants whose scheduled visits are due will be contacted telephonically prior to the appointment, and a text message will be sent to them a day prior to the appointment as a reminder. Regular review of participants´ communication logs will be done to assist in identifying study participants who potentially may pose to be a retention challenge or loss to follow-up. Re-emphasis on the importance of adhering to study visits and procedures will be conducted on these participants. The study team will also ensure each visit is done according to its scheduled time-point and visit-specific window period with aid of the study visit calculator. The use of these retention tools will help reinforce participant and study staff relationship assisting in study compliance and ensuring a positive study experience. Ensuring compensation for travel expenses and for the time lost during attendance as well as contacting them on special occasions such as Christmas, New Year, or similar culturally appropriate festivals where feasible, might help further to promote participant retention. The inclusion of participants’ representatives in the Community Advisory Board/Institutional meetings where study updates will be presented will also serve to reinforce adherence, retention and complete follow-up of the study participants. Tracing information will be documented in the communication log and information on discontinuation or deviation will be recorded in the participants file notes.

### Data management {19}

Electronic case report forms (eCRF) will be created for each participant and all study data collected will be entered into the eCRF. Some data may still be captured entirely or partially on paper source documents and will manually be entered into the eCRF. Accuracy and completeness of the data will be checked by monitoring visits at each site, and by pre-programmed edit checks that will flag out of range values.

Risk-based monitoring will be carried out according to the *monitoring plan*.

The sponsor will provide a framework for maintenance of quality in performance and reporting of laboratory procedures.

The study database will be locked after the data has been monitored by the sponsor and all queries issued through data cleaning activities have been completed and resolutions documented.

Essential documents will be retained until at least 2 years after the last approval of a marketing application in the International Conference on Harmonization (ICH) region and until there are no pending or contemplated marketing applications in an ICH region or at least 2 years have elapsed since the formal discontinuation of clinical development of the investigational product, or for not less than 10 years after trial completion, whichever is longer.

These documents should be retained for a longer period, however, if required by the applicable regulatory requirements or by an agreement with the sponsor. It is the responsibility of the sponsor to inform the investigator/institution as to when these documents no longer need to be retained.

### Confidentiality {27}

In the trial database and forms, participants will only be identified by a participant identification number, consisting of six figures, which represent the respective site and the enrollment number of the participant. The corresponding participant identification log will be kept in a securely locked separate trial site file, that only delegated staff will have access to. All participants´ records and laboratory specimen displaying names or addresses will be kept confidential in a secure storage area at the sites. The trial database will be encrypted, stored on secure servers with regular back-up, and access control.

### Plans for collection, laboratory evaluation and storage of biological specimens for genetic or molecular analysis in this trial/future use {33}

Genetic blood samples, stored for future testing, will be labelled using anonymous codes. Results of any genetic tests will not be disclosed to anybody not involved with the study.

## Statistical methods

### Statistical methods for primary and secondary outcomes {20a}

To establish an exposure-response model for DZD, the change in liquid culture MGIT time to positivity (TTP) will be modelled and linked to derived PK metrics. The model for TTP will be based on a previously develop and published model, linking a latent variable describing the decline in bacterial load to a model of probability of detection in MGIT (handling negative samples) and a time-to-event model for TTP [17]. The TTP value measured at baseline will be used to individualize the starting point for each participant. Only TTP values measured after start of treatment will be included in the fit of the model. Inter-individual variability with long-normal distributions will be included for parameters describing the bacterial load at baseline and the decline in bacterial load. The model may be adjusted as needed to achieve a satisfactory fit of the observed data. The fit will be evaluated primarily with two types of goodness of fit plots: visual predictive checks of the proportion of negative samples over time on treatment and Kaplan-Meier visual predictive checks of TTP per treatment week.

### Interim analyses {21b}

The data management and safety board (DSMB) will act as an advisory capacity to the Trial Steering Committee (TSC), to safeguard the interest of trial participants and to review the results of the interim analyses. It will also provide the TSC with recommendations on the continuation, premature closure of the trial or of single experimental treatment arms or extension of the study. The DSMB will meet at least every 6 months and more often if needed.

### Methods for additional analyses (e.g. subgroup analyses) {20b}

Descriptive summary statistics, such a mean/median of the time to culture conversion will be tabulated. Proportion of participants achieving culture conversion at each time point during treatment will be summarized.

A Cox regression model will be used to compare each arm with different DZD doses to the background regimen without DZD, censoring for death and loss to follow-up, to estimate the hazard ratio. The analysis will be adjusted for the baseline cultures using time to positivity (TTP) at the time of screening and enrolment. Demographic and clinical characteristics, such as gender, age, race, BMI, HIV status, smoking, and alcohol usage, will also be adjusted in sensitivity analyses.

### Methods in analysis to handle protocol non-adherence and any statistical methods to handle missing data {20c}

The primary analysis population is the intent-to-treat (ITT) analysis population. The ITT analysis population will consist of all randomized patients in the groups to which they were randomly assigned, and who have taken at least one dose of study treatment.

A secondary analysis will be of the adequate adherence (AA) analysis population. The AA analysis population will be the same as the ITT population with the following patients excluded: randomized patients not meeting the eligibility criteria; patients having missed 10 or more doses of their allocated treatment in the first 16 weeks of their treatment.

All safety analyses will use the safety analysis population: the safety analysis population will be defined as all patients who received any dose of study medication.

After entering the study data into the eCRFs, programmed database checks will raise automatic queries in case of any identified inconsistencies or incompleteness of the data. Further completeness and consistency checks will be performed by data management and any resulting queries will be sent through the database query system so as to leave an audit trail.

### Plans to give access to the full protocol, participant level-data and statistical code {31c}

The PanACEA consortium intends to make the protocol and dataset available, e.g. via the TB PACTS TB trials database.

## Oversight and monitoring

### Composition of the coordinating centre and trial steering committee {5d}

The TSC will be composed of at least 3 voting members, including a representative of the sponsor, the coordinator of the PanACEA consortium and an independent clinician.The role of the TSC is to provide overall supervision of the trial and ensure that the trial is conducted in accordance with Good Clinical Practice and Good Clinical Laboratory Practice principles. TSC meetings will be held on an ad hoc basis throughout the trial from first-participant-in to last-participant-out to evaluate participants’ safety. The TSC will formally report to the Sponsor. TSC specifics will be detailed and justified in the TSC charter.

### Composition of the data monitoring committee, its role and reporting structure {21a}

The DSMB will consist of five members: a clinician with experience in treatment for drug-sensitive and MDR-TB, an epidemiologist, a pharmacologist, a statistician and a TB laboratory science expert. The DSMB will be installed to safeguard the interest of trial participants and include an element of expert advice that is independent of the sponsor and the principal investigators. Further, the DSMB will review data and will make recommendations to the TSC to stop single arms or the whole trial if trial participation is an undue risk to participants.

### Adverse event reporting and harms {22}

All participants will be instructed during informed consent to report at any time any occurrence of AEs to the investigator. In addition, AEs will be solicited at every scheduled visit. The severity of AEs will be classified following the U.S. National Institutes of Health Common Terminology for Adverse Events 5.0 (CTCAE), available online at https://ctep.cancer.gov/protocoldevelopment/electronic_applications/docs/ctcae_v5_quick_reference_8.5x11.pdf, published November 27, 2017. An exception from this grading is from protocol version 2.1 onwards the prolongation of the QTcF interval. The severity in this case is graded according to Table 1. The deviation from the CTCAE severity grading is necessary due to the specific situation of TB participants; a population in whom a change in QTc over baseline is difficult to assess. Elevated heart rates at baseline are often due to disease, possibly elevated body temperature and/or anxiety after entering a trial. In a specific analysis of the Oflotub phase III study, it was confirmed that the elevated heart rates at baseline were associated with lower QTcF; and that specifically at baseline, QTcF correction undercorrects at these high heart rates [18]. Due to this limitation, there is a risk that an incorrect signal of QTcF prolongation over baseline in a participant will occur that in itself will not show a safety hazard to the participant but will result in lifesaving drugs being withheld. Therefore, the assessment of the severity of QTcF prolongation and the stopping of treatment in this study follows the ACTG A5343 phase 2 trial (the precedent of the above-mentioned trial); where a combination of BDQ and DLM was trialled and assessed for its potential to prolong the QT interval [14].

In this study, in order to prevent a false signal that might be due to a change in heart rate between assessments, a higher grade QTcF prolongation is defined as a combination of QTcF prolongation from baseline with an elevated absolute value, not a prolongation alone.

### Frequency and plans for auditing trial conduct {23}

The sponsor has created an audit plan that includes three audits performed by qualified auditors of partner institutions in the PanACEA consortium that take on trial-related responsibilities, and of subcontractors.

### Plans for communicating important protocol amendments to relevant parties (e.g. trial participants, ethical committees) {25}

Protocol amendments, after being fully approved by applicable ethics committees and regulatory agencies, will be transmitted to investigators and a protocol amendment training will be performed and documented.

### Dissemination plans {31a}

Trial outcomes will be important for TB participants and their treating healthcare providers. The results of this trial will be disseminated via scientific publications through high-impact, international, peer-reviewed journals and through scientific conferences; open access schemes will be used.

## Discussion

The occurrence of COVID-19 during trial preparation affected IMP production, and COVID-19 in trial participants may generate false safety signals if attributed to the trial drugs. Therefore, we included guidance on COVID-19 testing based on symptoms or hypoxemia into the trial-specific manuals and discussed preventive infection control measures with the study sties. Furthermore, to enable on-site monitoring during international lockdowns, we contracted local monitors instead of relying on international travel.

### Trial status

At the time of writing this publication, the protocol version 2.1 was used in South Africa and protocol version 2.0 in Tanzania. Recruitment started at the end of October 2021 and is expected to end in Q3 2022.

## Data Availability

All parties conducting the trial will have access to the final trial dataset.

## Abbreviations

AE: Adverse event
ALT: Alanine aminotransferase
ARV: Antiretroviral
ART: Antiretroviral treatment
AST: Amino aspartate transferase
AV: Atrioventricular
BDQ: Bedaquiline
BP: Blood pressure
DLM: Delamanid
DOT: Directly observed treatment
DSMB: Data management and safety board
DZD: Delpazolid
EBA: Early bactericidal activity
ECG: Electrocardiogram
eCRF: Electronic case report forms
FDA: Food and Drug Administration
FQ: Fluorquinolone
HIV: Human immunodeficiency virus
ICH: International Conference on Harmonization
IMP: Investigational medicinal product
MAO: Monoamine oxidase
MAO A: Monoamine oxidase A
MDR-TB: Multidrug-resistant tuberculosis
MTB: Mycobacterium tuberculosis
MXF: Moxifloxacin
LZD: Linezolid
PK: Pharmacokinetic
PTM: Pretomanid
QTcF: Fridericia corrected QT interval
RR-TB: Rifampicin-resistant TB
TB: Tuberculosis
TTP: Time to positivity
TSC: Trial Steering Committee
XDR-TB: Extensively drug-resistant tuberculosis
